# Floor Maze Test is capable of differentiating spatial navigation between frail and pre-frail institutionalized older persons

**DOI:** 10.1590/1980-5764-DN-2022-0070

**Published:** 2023-07-17

**Authors:** Eric Hudson Evangelista e Souza, Luana Lemos Leão, Alfredo Maurício Batista de Paula, Vinícius Dias Rodrigues, Andréa Camaz Deslandes, Jerson Laks, Renato Sobral Monteiro

**Affiliations:** 1 Universidade Estadual de Montes Claros, Programa de Pós-Graduação em Ciências da Saúde, Montes Claros MG, Brazil.; 2 Universidade Federal do Rio de Janeiro, Instituto de Psiquiatria, Rio de Janeiro RJ, Brazil.; 3 Universidade Federal Fluminense, Programa de Pós-Graduação em Neurologia/Neurociência, Niterói RJ, Brazil.

**Keywords:** Frailty, Spatial Navigation, Cognitive Aging, Functional Residual Capacity, Fragilidade, Navegação Espacial, Envelhecimento Cognitivo, Capacidade Residual Funcional

## Abstract

Investigation of the association between physical frailty and cognitive performance through spatial navigation is important to enable the identification of individuals with cognitive impairment and physical comorbidity.

**Objective:**

To analyze the association between spatial navigation and frailty in frail and pre-frail institutionalized older adults.

**Methods:**

Forty older people of both sexes, aged 60 years or over, residing in four Brazilian Long-Term Care Facilities (LTCFs) participated in this study. The following tests were applied: Mini-Mental State Examination (MMSE), 2.44m Timed Up and Go, Floor Maze Test (FMT), and Fried's frailty criteria. For data analysis, the Mann-Whitney and independent t-tests were used to compare the groups (frail x pre-frail), principal component analysis was used to explore the main variables related to the data variance, and binary logistic regression to estimate associations.

**Results:**

There was a significant difference in performance in the FMT immediate maze time (IMT) (p=0.02) and in the delayed maze time (DMT) (p=0.009) between the pre-frail and frail older adults. An association between FMT DMT performance and frailty was found, showing that older people with shorter times on the DMT (better performance) had approximately four times the chance of not being frail (odds ratio – OR=4.219, 95% confidence interval – 95%CI 1.084–16.426, p=0.038).

**Conclusion:**

Frailty is associated with impaired spatial navigation ability in institutionalized older adults, regardless of gait speed performance.

## RESUMO.

A investigação da associação entre fragilidade física e desempenho cognitivo por meio da navegação espacial é importante para possibilitar a identificação de indivíduos com déficit cognitivo e comorbidade física.

**Objetivo:**

Analisar a associação entre navegação espacial e fragilidade em idosos institucionalizados frágeis e pré-frágeis.

**Métodos:**

Participaram deste estudo 40 idosos de ambos os sexos, com idade igual ou superior a 60 anos, residentes em quatro instituições de longa permanência (ILPI) brasileiras. Foram aplicados os seguintes testes: Miniexame do Estado Mental (MEEM), 2,44m Timed Up and Go, Floor Maze Test (FMT) e os critérios de fragilidade de Fried. Para a análise dos dados, foram utilizados os testes *t* independente e de Mann-Whitney para comparar os grupos (frágeis x pré-frágeis), foi feita análise de componentes principais para explorar as principais variáveis relacionadas à variância dos dados e regressão logística binária para estimar associações.

**Resultados:**

Houve diferença significativa no desempenho do FMT no tempo imediato do labirinto (TIL) (p=0,02) e no tempo posterior do labirinto (TPL) (p=0,009) entre os idosos pré-frágeis e frágeis. Encontrou-se associação entre o desempenho no FMT TPL e fragilidade, mostrando que idosos com menor tempo de TPL (melhor desempenho) tiveram aproximadamente quatro vezes mais chance de não serem frágeis (*odds ratio –* OR=4,219, intervalo de confiança de 95% – IC95% 1,084–16,426, p=0,038).

**Conclusão:**

A fragilidade está associada à habilidade de navegação espacial prejudicada em idosos institucionalizados, independentemente do desempenho da velocidade da marcha.

## INTRODUCTION

The frailty syndrome is an age-related condition that is characterized by a decrease in physiological reserves and compromised responses to chronic stress, impairing the body's capacity to cope with adverse health outcomes^1–4^. Frail individuals are those who require the most care, which makes frailty an important marker for managing elderly health conditions^5^.

According to the scientific community, Fried et al.^6^ propose the most accepted conceptual model of frailty^6^. The “Frailty Phenotype” described by Fried et al.^6^ establishes that exhaustion, dynapenia, reduced gait speed, involuntary weight loss, and low levels of physical activity are substantial criteria for determining frailty. The frail state reduces the individual's independence and increases the chances of institutionalization, hospitalization, and/or early death^2–4,7,8^.

Currently, gait speed, which is a criterion for determining frailty, stands out among these markers and its use is recommended as there is an association between slow gait and the onset of Alzheimer's disease (AD) and future dementia^9–11^. The association between frailty and oxidative stress helps to understand the increased risk of frail older people to present AD^12,13^.

Studies report that the severity of the frail state is associated with an accelerated global cognitive decline and predicts mild cognitive impairment (MCI)^14,15^. A 12-year cohort study found that frailty, after adjusting for age, sex and education, is associated with a high risk of developing MCI, so that each one-unit increase in frailty was associated with a 63% increase in the risk of developing MCI (adjusted odds ratio – OR_adj_=1.63; 95% confidence interval – 95%CI 1.27–2.08)^15^.

Among the cognitive domains affected in institutionalized older adults is the spatial navigation ability^16^, which is characterized by the integration of complex cognitive processes, such as visual perception, learning, memory, and executive functions^16–18^. The hippocampus, parahippocampal gyrus, prefrontal, parietal and entorhinal cortex are the main brain regions triggered by spatial navigation ability and are sensitive to normal aging^19,20^.

The Floor Maze Test is an instrument for measuring spatial navigation ability, executive functions, and memory, which has been applied to institutionalized older adults for cognitive screening^16,21^. Among the reasons for choosing this instrument are its ease of application, low cost, and reliability of results^21^. Verghese et al.,^22^ in a longitudinal study, used the FMT to assess spatial navigation and found that the 10-second increment in FMT execution time is related to 25% increase in the risk of cognitive decline and 53% increase in the risk of motoric cognitive impairment, being a state of prodromal dementia. Some investigations have identified possible hyperintensities in the cerebral microvasculature arising from frailty in important regions for spatial navigation, such as the hippocampus^23,24^. This finding strengthens the original definition of cognitive frailty as a condition of poor cognitive performance related to physical conditions in the absence of apparent neurodegenerative diseases.

Investigating the association between frailty and cognitive performance through spatial navigation is important to enable the early identification of individuals with physical comorbidity and cognitive impairment simultaneously. From this perspective, the verification of a single test capable of discriminating pre-frail and frail older adults, and at risk of cognitive decline, becomes relevant for screening for prodromal dementia, which would facilitate the implementation of more specific interventions to prevent and/or minimize the development of dementia as well as adverse events arising from the frail condition^25^. To date, we have not identified studies that have spatial navigation and its interaction with frailty. In this context, the present study aimed to analyze the association between spatial navigation and frailty in pre-frail and frail institutionalized older adults.

## METHODS

### Study design and ethical approval

This cross-sectional study was performed in the Brazilian cities of Rio de Janeiro and Montes Claros. The data is part of two research projects, which can be accessed at the Brazilian Registry of Clinical Trials (http://www.ensaiosclinicos.gov.br), protocols RBR-6rytw2 and RBR-8dv3kg. Both projects were approved by the Research Ethics Committees of Universidade Federal Fluminense (1,178,067/2015) and Universidade Estadual de Montes Claros (2,398,863/2017). All participants or their legal guardians signed an informed consent form.

### Participants

A finite population of 200 older people living in four Brazilian Long-Term Care Facilities (LTCFs) was included, three of these located in the city of Rio Janeiro (RJ) and one in Montes Claros (MG). The sample calculation, considering the significance level of 5%, the proportion of 50% of individuals per group, and the confidence interval of 95%, was performed using the Australian Bureau of Statistics website (https://www.abs.gov.au/websitedbs/D3310114.nsf/home/Sample+Size+Calculator), which identified that the sample should be composed of 132 individuals. Participants were recruited according to the following inclusion criteria:

- Being a resident of a long-stay institution for older adults;
- Having preserved communication skills;
- Having medical consent to perform physical exertion;
- Not having neurological impairment or neurodegenerative diseases, based on the diagnosis made by the physician.

The following exclusion criteria were applied:

- Having severe cardiovascular impairment;
- Suffering from acute musculoskeletal injury and
- Delirium.

Only 40 older people of both sexes, aged over 60 years, were considered eligible to participate in this study. The reduction in the number of participants was due to the high prevalence of cognitive impairment, dementia, and physical disability in LTCFs^26^; in addition, some older adults were unable to complete the assessments, which forced us to use convenience sampling, since the number of participants was below that was estimated in the sample calculation.

In the morning, the older adults underwent the cognitive test (Mini-Mental State Examination — MMSE) and, in the afternoon, on a different day from the cognitive test, they performed the frailty tests. The Floor Maze Test (FMT) was applied in the afternoon. The main aspects observed were: older adults’ willingness to perform activities, meal times, and free time to answer questionnaires and perform physical tests.

### Global cognitive assessment

The MMSE was used to assess global cognition^27^. It consists of 11 items with a 30-point score and was used to track the cognitive level through memory and short-term retrieval, temporal and spatial orientation, language and visuospatial skills, calculus, and praxis. Different reference values were used for different levels of education: ≤ 13 points (illiterate), ≤18 points (from one to seven years of schooling), and ≥26 points (>eight years of schooling). There was no exclusion of participants based on the stratification of the MMSE.

### Spatial navigation assessment

Spatial navigation was assessed using the FMT^21^. The evaluation consisted of two moments, with a 10-minute interval between the end of the first and the beginning of the second. The time spent between the end of instruction and discovery of the correct route (planning time — PT) and the time spent traveling from the entrance to the exit of the maze (immediate maze time — IMT) were recorded. After the end of the course, the individuals were taken to a room, without visual contact with the maze, where they remained for 10 minutes. Then they were positioned at the entrance of the maze and instructed to follow the path that leads to the exit (delayed maze time — DMT) without prior planning. The amount of time used to perform the IMT and DMT is inversely related to the spatial navigation capacity; in this sense, the longer the time used by the IMT and DMT participant, the worse the spatial navigation ability. In this study, PT was not used, as there was no guarantee that the older adults really planned the route.

### Frailty assessment

The frailty syndrome was evaluated using the Frailty Phenotype proposed by Fried et al.,^6^ which considers five objectively measurable components: unintentional weight loss, self-reported exhaustion, low level of physical activity, slow gait (time spent walking 4.57 m), and grip strength (hydraulic hand dynamometer SH5001 SAEHAN^®^). The older adults were classified as pre-frail (one or two components) and frail (three or more components). No robust older individuals were identified.

### Gait speed assessment

The 2.44 m Up and Go test was selected to assess gait through agility and dynamic balance. Test execution time is timed from the sound signal emitted by the evaluator to the moment when the subject sits on the chair. After a familiarization test, three tests were applied and the shortest time was counted^28^.

### Data analysis

Descriptive statistics were used and the data are presented as mean and standard deviation (data with normal distribution), median, minimum and maximum (data with non-normal distribution). The Shapiro-Wilk test was used to test normality in the distribution of data regarding age, Timed Up and Go, FMT (IMT and DMT), and MMSE. After this analysis, the Mann-Whitney test was chosen to verify differences in age, agility, and dynamic balance, and time of IMT and DMT between groups. The independent t-test was used to compare the mean values of global cognition. Principal component analysis (PCA) was used to explore the variance and possible associations between variables (IMT, DMT, MMSE, age, and gait) within the sample. The results of this analysis included the combination of the two main generated components, which explained 72% of the variability present in the data. The variables that presented the highest values in the two main components were used in a binary logistic regression model, in order to test the association of these variables with frailty, using odds ratio estimates. The Backward Stepwise method was used and the pre-frail stage was considered as a reference category. Omnibus, R² Cox and Snell, and R² Nagelkerke tests were used to test the coefficient of each model. p-values ≤0.05 were considered as indicators of statistical significance in all analyses, which were performed using Statistical Package for the Social Sciences (SPSS) version 21.0 and Jamovi (version 1.6).

## RESULTS

**Figure 1:**
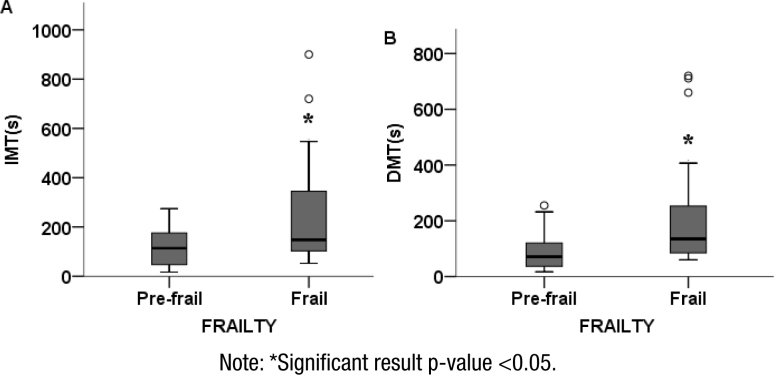
Performance on immediate maze time and delayed maze time between groups. (A) Values of immediate maze time and (B) values of delayed maze time.

Table 1 shows the comparison of age, Up and Go test, IMT, DMT, and MMSE between groups. There was no statistical difference regarding age (p=0.541) and gait (p=0.055) between groups. However, there was a difference in performance between pre-frail and frail individuals in IMT (p=0.02) and in DMT (p=0.009), revealing that frail older adults take longer to go through the maze, both in IMT and in the DMT, which shows that such individuals have compromised spatial navigation abilities. A comparison of the groups’ IMT and DMT times through the median and interquartile ranges is available in Figure 1. The independent t-test revealed that the pre-frail older adults had superior global cognition when compared to frail ones (p=0.004), indicating that the former have the most preserved global cognition.

**Table 1:** Comparison between age, Timed Up and Go test, immediate maze time and delayed maze time, and Mini-Mental State Examination.

Variables	Group	n	Descriptive data*	Test
Age (years)	Pre-frail	21	84 (68-93)	U=177.00
	Frail	19	84 (69-93)	
Up and Go	Pre-frail	21	12 (6-44)	U=105.50
	Frail	19	17 (10-27)	
IMT_(s)_	Pre-frail	21	110 (17-274)	U=114.50†
	Frail	19	141 (53-900)	
DMT_(s)_	Pre-frail	19	71 (17-255)	U=79.00‡
	Frail	17	135 (60-720)	
MMSE_(score)_	Pre-frail	21	23,68±4,59	t=3.06†
	Frail	19	17,35±6,94	

Abbreviations: IMT: immediate maze time; DMT: delayed maze time; MMSE: Mini-Mental State Examination; U: Mann-Whitney test; t: independent t-test. Notes:

* Descriptive data are presented in median, maximum and minimum values, mean and standard deviation.

† Significant result p-value <0.05;

‡ Significant result p-value <0.01

The PCA based on the correlation matrix was used to standardize the data through the Z-score and prevent the variables with a larger numerical scale (IMT and DMT) from gaining greater importance over the other dependent variables. The analysis revealed that the combination of the two main components explained a total of 72.2% of the data variance, with the first component contributing 47.61% and the second 24.63% in the model. The variables that most contributed to the first component were IMT and DMT, and, to the second, age and gait speed.

Table 2 presents the association between the variables IMT, DMT, age, gait, and frailty. The binary logistic regression model containing the frailty variable and the independent variables (IMT, DMT, age, and gait) was significant, showing that the DMT was the only variable associated with frailty (Omnibus coefficient test=8,718, degrees of freedom (df)=1, p<0.03). The Hosmer-Lemeshow test showed a high goodness of fit (χ^2^=0.313, df=1, p=0.576). The variable explained 22–29.5% of the frailty (Cox and Snell R^2^=0.220 and Nagelkerke R^2^=0.295). The older adults who showed the lowest performance times in the DMT (best performance) had four times the chance of belonging to the pre-frail group. The regression model before considering the independent variables showed 54.3% of correct prediction. After including the DMT variable, the predictive power of the model increased to 65.7%. Sensitivity and specificity were 52.6 and 81.3%, respectively.

**Table 2:** Association between immediate maze time, delayed maze time, age, gait speed and frailty.

Dependent variable — frailty (pre-frail* and frail)						
	Variable	B	Wald	p-value	OR	CI
Step 1	IMT	-0.446	0.214	0.644	0.640	0.097–4.233
	DMT	2.034	3.908	0.048	7.643	1.018–57.395
	Age	-0.670	2.027	0.154	0.512	0.204–1.287
	GAIT	-0.012	0.001	0.973	0.988	0.483–2.019
Step 2	IMT	-0.453	0.235	0.628	0.635	0.102–3.978
	DMT	2.037	3.954	0.047	7.667	1.030–57.105
	Age	-0.674	2.208	0.137	0.510	0.210–1.240
Step 3	DMT	1.737	4.599	0.032	5.683	1.161–27.805
	Age	-0.603	1.984	0.159	0.547	0.236–1.266
Step 4	DMT	1.440	4.309	0.038	4.219	1.084–16.426

Abbreviations: IMT: immediate maze time; DMT: delayed maze time; B: unstandardized coefficients; OR: odds ratio; CI: confidence interval.

* Note: reference category.

## DISCUSSION

This study showed that execution time in the DMT can be associated with the frailty stages. Older adults who presented shorter execution times in the DMT (better performance) have more preserved spatial navigation abilities and are more likely to belong to the pre-frail group.

Although frailty is an age-dependent clinical condition^29,30^, there was no difference in the distribution of age between the groups, as well as no association with frailty in this study. The sample size may be a possible explanation for this divergence. Regarding space navigation, Sanders et al.^21^ reported that the performance of individuals in both IMT and DMT was not associated with age, which leads us to believe that frailty can be an influencing factor that does not depend on age in spatial navigation ability.

A previous study^31^ demonstrated that the association between frailty and cognitive performance can be found from 75 years onwards (OR_adj_=2.78, 95%CI 1.23–6.97) and its chance is 15 times greater from 85 years old (OR_adj_=15.62, 95%CI 2.20–110.99). In our study, the screening of global cognition between groups showed a significant difference, which may be a confounding factor to be considered; however, this difference was also found in studies that compared global cognition and cognitive performance between pre-frail and frail older adults^31–34^. Alves et al.^32^ showed that global cognition and short-term memory are different between pre-frail and frail older adults (p<0.01), with global cognition explaining 14–19% of the frailty model.

Although global cognition was different between groups, in the PCA analysis this variable did not show sufficient value to compose the binary logistic regression model, showing the direct association of frailty with spatial navigation.

Some studies report an association between spatial navigation, global cognition, and cognitive impairment^16,17^. Tangen et al.^17^ verified that individuals with subjective cognitive impairment, MCI, and AD had worse performance in the FMT, according to the severity of the impairment. Zanco et al.^18^ showed that older people with AD have significant deficits in spatial navigation and that IMT and DMT are related to the impairment of global cognitive status. Our results showed that time spent to go through the maze in both IMT and DMT was different between groups. Frail older adults took longer to exit the maze, which corroborates previous studies, as these individuals also had lower levels of global cognition.

It is important to emphasize that only DMT was associated with the stages of frailty in the binary logistic regression analysis. A possible explanation is the fact that gait speed is only associated with DMT^21^ and is one of the components for the assessment of frailty^7^; in addition, worse performance on DMT is associated with an increased risk of developing motoric cognitive impairment^22^. Our data reinforce this sensitivity, as frail individuals have worse gait performance^35^, and a better performance in DMT was associated with the pre-frail stage. Time on IMT and age were considered protective for the pre-frail stage in our analysis, which is intuitive, as better IMT performance and younger age are associated with more preserved cognition and lower risk of motoric cognitive impairment and frailty^17,21,22,36^. Such information provides us with guidance that should be explored in further studies that investigate the association between spatial navigation and frailty through the FMT.

Although questions related to gait are considered important in understanding frailty, cognition, and risk of dementia^9–11^, there was no difference between groups or association with frailty. The choice of the Up and Go test as a gait speed evaluation parameter was determined by the similarity of its execution with the FMT since both tests require a response to an initial sound command and a change of direction during the trajectory.

A close interdependent relationship between frailty and cognition is also described in the literature, postulating that the impairment of one component can interfere with the other, a cycle of deleterious events that reduce functional capacity and quality of life, which may increase the risk of dementia and evolution to death^37^. To date, the mechanisms and pathophysiology underlying the increased risk of dementia in frail older people are not completely clear. However, frailty and dementia are complex and heterogeneous conditions that share risk factors in their development and may present a pathophysiological convergence concerning the brain^12^.

The study by Chen et al.^23^ using magnetic resonance identified that the presence of frailty verified through Fried's criteria^6^ is related to signatures in the brain regions that are fundamental for spatial navigation, such as a reduction in the volume of the hippocampus, middle frontal gyrus, right inferior parietal lobe and middle occipital gyrus, which may explain the poorer performance of frail older people in the ability of spatial navigation found in our study.

Both spatial navigation and frailty seem to be dementia predictors^12,21^. Our results demonstrate that the FMT, in addition to being a predictor of MCI, motor deficit, and a predictor of prodromal dementia^9,16,17^, is a practical test, of easy clinical application and low cost,^21^ that can be used to differentiate from pre-frail and frail older adults through spatial navigation. It facilitates the diagnosis of cognitive decline and frailty, which are two important conditions related to the health of institutionalized older adults.

Our study has some important limitations; the first is sample size. It was not possible to reach the number of individuals predicted in the sample size calculation. Another limitation is the absence of robust older people in the sample. With a larger sample and the presence of robust older adults, it would be possible to further explore the data, to establish the magnitude of the influence of independent variables on dependent ones and the degree of accuracy in differentiating pre-frail and frail older adults. Although our study has limitations, the results of our analysis point to an interesting path that should be investigated in longitudinal studies with larger samples in order to identify individuals with simultaneous cognitive and physical impairment at an early stage.

Frailty is associated with impaired spatial navigation ability in institutionalized older people regardless of age and gait speed. Institutionalized pre-frail older people perform better on the FMT when compared to their frail peers. The early identification of frailty and cognitive impairment has a substantial impact on the quality of life of older adults, as it allows the use of multicomponent methods and treatments, such as increased exercise and cognitive training, in order to reverse the frail state, improve the cognitive performance and delay and/or prevent the onset of dementia. In addition, early identification of dementia and frailty is extremely important. Using one test to identify both conditions reduces the number of tests used to evaluate older adults, reduces costs, and facilitates the adoption of health policies for this target population. It is important to prioritize multi-component strategies that integrate financial investments and quality of life, focusing on frail older adults and actions aimed at them.
